# Calcitonin gene-related peptide receptor antagonist BIBN4096BS regulates synaptic transmission in the vestibular nucleus and improves vestibular function via PKC/ERK/CREB pathway in an experimental chronic migraine rat model

**DOI:** 10.1186/s10194-022-01403-1

**Published:** 2022-03-08

**Authors:** Ruimin Tian, Yun Zhang, Qi Pan, Yunfeng Wang, Qianwen Wen, Xiaoping Fan, Guangcheng Qin, Dunke Zhang, Lixue Chen, Yixin Zhang, Jiying Zhou

**Affiliations:** 1 Department of Neurology, The First Affiliated Hospital of Chongqing Medical University, 1st You Yi Road, Yu Zhong District, 400016 Chongqing, China; 2 Department of Neurology, Nanchong Central Hospital, Nanchong, China; 3 Laboratory Research Center, The First Affiliated Hospital of Chongqing Medical University, Chongqing, China

**Keywords:** CGRP, Migraine, Vestibular dysfunction, Vestibular nucleus, Central sensitization, Synaptic transmission

## Abstract

**Background:**

Vestibular symptoms are frequently reported in patients with chronic migraine (CM). However, whether vestibular symptoms arise through overlapping neurobiology of migraine remains to be elucidated. The neuropeptide calcitonin gene-related peptide (CGRP) and CGRP1 receptor play important pathological roles in facilitating central sensitization in CM. Therefore, we aimed to investigate whether CGRP1 receptor contributes to vestibular dysfunction after CM by improving synaptic transmission in the vestibular nucleus (VN).

**Methods:**

A CM rat model was established by recurrent intermittent administration of nitroglycerin (NTG). Migraine- and vestibular-related behaviors were assessed. CGRP1 receptor specific antagonist, BIBN4096BS, and protein kinase C (PKC) inhibitor chelerythrine chloride (CHE) were administered intracerebroventricularly. The expressions of CGRP and CGRP1 receptor components, calcitonin receptor-like receptor (CLR) and receptor activity modifying protein 1 (RAMP1) were evaluated by western blot, immunofluorescent staining and quantitative real-time polymerase chain reaction in the vestibular nucleus (VN). Synaptic associated proteins and synaptic morphological characteristics were explored by western blot, transmission electron microscope, and Golgi-cox staining. The expressions of PKC, phosphorylated extracellular signal regulated kinase (p-ERK), phosphorylated cAMP response element-binding protein at serine 133 site (p-CREB-S133) and c-Fos were detected using western blot or immunofluorescent staining.

**Results:**

The expressions of CGRP, CLR and RAMP1 were significantly upregulated in CM rats. CLR and RAMP1 were expressed mainly in neurons. BIBN4096BS treatment and PKC inhibition alleviated mechanical allodynia, thermal hyperalgesia and vestibular dysfunction in CM rats. Additionally, BIBN4096BS treatment and PKC inhibition markedly inhibited the overexpression of synaptic associated proteins and restored the abnormal synaptic structure in VN after CM. Furthermore, BIBN4096BS treatment dysregulated the expression levels of PKC, p-ERK and p-CREB-S133, and attenuated neuronal activation in VN after CM.

**Conclusions:**

The present study demonstrated that CGRP1 receptor inhibition improved vestibular function after CM by reversing the aberrant synaptic transmission via downregulating PKC/ERK/CREB signaling pathway. Therapeutic interventions by inhibiting CGRP/CGRP1 signaling may be a new target for the treatment of vestibular symptoms in CM.

## Introduction

Patients with migraine usually experience not only headache but also balance problems and dizziness, i.e. motion sickness and recurrent vertigo [1, 2]. With headache frequency increased, the incidence of dizziness and associated handicap significantly elevated [2]. Of note, up to 85% of patients with chronic migraine (CM) reported vestibular symptoms [2]. Unfortunately, most migraine patients with vestibular symptoms do not respond well to treatment with traditional vestibular suppressants [3]. Despite this, it is not yet understood whether vestibular symptoms arise through overlapping neurobiology or independent mechanisms of migraine. Consequently, the application of migraine-specific treatments remains limited in this population [4]. Central sensitization of trigeminovascular neurons in the trigeminal nucleus caudalis (TNC) is a leading culprit that contributes to sensory hypersensitivity in migraine [5], while the enhancement of synaptic transmission is the neural basis of central sensitization in rat models of CM [6, 7]. Our previous study demonstrated that central sensitization in the vestibular nucleus (VN) was the potential mechanism for vestibular symptoms in migraine, and vestibular dysfunction was significantly related to neuronal activation in VN [8]. Thus, effectively suppressing neuronal activation via modulating synaptic transmission efficiency in VN might be a promising approach in the treatment of vestibular symptoms in migraine patients.

The neuropeptide calcitonin gene-related peptide (CGRP) has been demonstrated to play an essential role in facilitating pain transmission [9]. CGRP is a 37-amino acid neuropeptide, and produced mainly by neurons [10]. CGRP mediates its function primarily by activating CGRP1 receptor, containing calcitonin receptor-like receptor (CLR), a G protein-coupled receptor (GPCR), together with receptor activity modifying protein 1 (RAMP1), a single transmembrane protein which is required for functional expression of CLR at the cell surface [11, 12]. Prior study found that the expression level of CGRP in VN increased significantly in a rat model of motion sickness, whilst anisodamine could markedly suppress the CGRP expression in VN and reverse motion sickness-related behaviors [13]. Our previous study showed similar results in CM rats, and blunting CGRP synthesis in VN caused a marked reversal of nitroglycerin (NTG)-induced vestibular dysfunction [8]. To date, clinical trials targeting CGRP and its receptors witness a great success in migraine treatment [14]. However, whether CGRP and its receptors are viable therapeutic targets in migraine patients with vestibular symptoms is uncertain, and whether CGRP antibodies and CGRP1 receptor antagonists can alleviate vestibular symptoms in migraine patients requires further investigation.

CGRP has been demonstrated to produce long-term potentiation (LTP) in the insular cortex and anterior cingulate cortex [15]. This phenomenon could be significantly blocked by specific CGRP1 receptor complex antagonists, CGRP_8 − 37_ or BIBN4096BS, which further achieving analgesic effects [15, 16]. Besides that, these two antagonists could also reverse pain-related synaptic function in the amygdala of arthritic rats, as evidenced by the reduced neuronal excitability and decreased amplitude of miniature excitatory postsynaptic currents (mEPSCs) [17], pointing that CGRP and CGRP1 receptor signaling might modulate synaptic function in CM. Although CGRP1 receptor is generally considered a Gα_s_-coupled GPCR, it is becoming clear that it also couples to additional G proteins and other proteins [12, 18]. In vitro studies showed that CGRP activated protein kinase C (PKC) and extracellular signal regulated kinase (ERK) in HEK293 cells, and pre-incubation spinal cord slices with specific ERK or PKC inhibitor significantly shortened CGRP-stimulated firing time of lamina I neurons [19]. Moreover, cAMP response element-binding protein (CREB), one of the major downstream mediators of ERK, has been considered as an important indicator of central sensitization in CM rats [20, 21]. Downregulation of phosphorylated CREB at Serine 133 site (p-CREB-S133) was significantly associated with the reduction of neuronal activation in TNC after CM [21]. These data suggests that CGRP and CGRP1 receptor modulate synaptic transmission in CM rats might through PKC/ERK/CREB signaling pathway.

Based on the above-mentioned evidence, we hypothesized that CGRP1 receptor antagonist BIBN4096BS would reduce neuronal activation in VN and alleviate vestibular dysfunction in rat models of CM and that these beneficial effects might be mediated by PKC/ERK/CREB signaling. Through these attempts, we explore the therapeutic efficacy of CGRP1 receptor antagonist in a CM model as a preclinical approach for potential clinical translation in migraine patients with vestibular symptoms.

## Materials and methods

### Animals

All experiments were approved by the Commission of Chongqing Medical University for ethics of experiments on animals and followed the National Institutes of Health Guide for the Care and Use of Laboratory Animals. All animal experiments complied with the ARRIVE guidelines. One-hundred and eight male Sprague Dawley (SD) rats weighing 250–300 g were obtained from Hunan Slack Jingda Experimental Animal Co., Ltd. (Hunan, China). They were kept in the standard condition of 12 h light-dark cycle with the temperature at 23℃ ± 2 ℃ and 50 ± 5% relative humidity of the environment. Water and food were adequately provided. The rats were randomly assigned to each group after a week of acclimatization.

### Establishment of the chronic migraine model

The general procedure for inducing CM in male SD rats via nitroglycerin (NTG) injection was performed as previously described [8]. In brief, 5 mg/ml NTG (Beijing Regent, China) that was dissolved in 30% alcohol, 30% propylene glycol and water was prepared as stock solution. Prior to each injection, NTG stock solution was diluted to 1 mg/ml with 0.9% saline for injection use. All rats were randomly received intraperitoneal injection of 10 mg/kg of NTG or 0.9% saline at an equal volume every other day for 9 days (five times in total). According to the previous study, the mechanical thresholds were comparable between rats that received 0.9% saline and 6% propylene glycol + 6% alcohol + 0.9% saline [22]. After the fifth injection, rats were then placed back to the cages until sacrifice.

### Drug administration

BIBN4096BS (MedChemExpress, HY-10,095 A), a specific CGRP1 receptor antagonist, and chelerythrine chloride (CHE) (MedChemExpress, HY-12,048), a PKC inhibitor, were injected into the left lateral ventricle by intracerebroventricular administration as described previously [23]. BIBN4096BS was dissolved in 10% DMSO, 40% PEG300, 5% Tween80 and 45% saline to a final concentration of 0.01 µg/µl, which was administered at a dose of 0.1 µg/day/rat 2 h before each NTG injection based on previous study [24]. The CHE was dissolved in the same solution as BIBN4096BS to a final concentration of 1 mmol/L, which was administered at a dose of 10 nmol/day/rat based on previous study [25]. And the CHE was intracerebroventricularly injected same as the BIBN4096BS administration route, that is, injected 2 h prior each NTG administration. An equivalent volume of solvent (10 µl) was administered intracerebroventricularly as a corresponding vehicle control group (CM + vehicle).

### Stereotaxic surgery procedures and intracerebroventricular injection

As described previously, rats were anesthetized with 10% chloral hydrate (4 ml/kg, intraperitoneal) combined with analgesics (0.01 mg/kg buprenorphine) [8]. When the pedal reflex was absent, they were fixed in a stereotactic frame (ST-51,603; Stoelting Co, Chicago, IL, USA) with a heating pad. The skin was incised on the longitudinal plane to expose the bregma. A cranial burr hole with a diameter of 1 mm was performed above the left dura at the following coordinates aiming for the left lateral ventricle: 1.0 mm rear from the bregma and 1.5 mm left lateral to the bregma. A sterile cannula with a screw cap was implanted at the above coordinates and stabilized with dental cement [21]. The animals were allowed to recover for at least 7 days until their thermal and mechanical pain thresholds returned to the preoperative levels.

Intracerebroventricular injections were performed in anesthetized rats using a 10-µl Hamilton syringe through the cannula at following stereotaxic coordinates: 1.0 mm rear from the bregma, 1.5 mm left lateral to the bregma, and 4.0 mm from the skull plane. Before and after each injection, the patency of the guide cannula was checked. The infusion was performed at a rate of 2 µl/min. After infusion, the needle was left in place for additional 10 min and then was withdrawn over 1 min. Next, animals were placed back to the heating pads for recovery and subsequent NTG injection.

### Experimental design and animal groups

#### Experiment 1

To evaluate endogenous expression levels of CGRP and CGRP1 receptor components (CLR and RAMP1), rats were randomly divided into two groups to perform western blot or quantitative real-time polymerase chain reaction (qPCR) and immunofluorescence staining: Saline and CM (n = 6/group). Rats were subjected to behavioral tests 15–20 min prior and 2 h post NTG injection. And the basal vestibular function test was performed on the day before NTG administration. In addition, the cellular co-localizations of CLR and RAMP1 were evaluated using double immunofluorescence staining in Saline and CM group (n = 3/group).

#### Experiment 2

The neuroprotective effects of CGRP1 receptor antagonist treatment (BIBN4096BS) for CM were evaluated. Rats were randomly divided into four groups: Saline, CM, CM + vehicle and CM + BIBN4096BS (n = 6/group). Behavioral tests were performed 15–20 min prior intracerebroventricular injection and 2 h post NTG injection, and then sacrificed to collect VN tissues for western blot, qPCR or immunofluorescence staining to evaluate the expression levels of CGRP, CLR, RAMP1 and synaptic associated proteins (post-synaptic density protein 95: PSD95; synaptophysin: Syp; synaptotagmin-1: Syt-1), for transmission electron microscope scanning to evaluate the thickness of postsynaptic density (PSD), length of synaptic activity zone and synaptic interface curvature, and for Golgi-cox staining to evaluate dendritic spines density in VN.

#### Experiment 3

To assess the potential molecular pathway of CGRP1 receptor in anti-neuronal activation effect, the specific PKC inhibitor, CHE, was administered to CM rats. The groups included Saline, CM, CM + vehicle, CM + BIBN4096BS and CM + CHE (n = 6/group). The administration route of BIBN4096BS and CHE was same as that in Experiment 2. Behavioral tests were performed 15–20 min prior intracerebroventricular injection and 2 h post NTG administration. The VN tissues were collected for western blot to measure protein levels of PKC, phosphorylated ERK, phosphorylated CREB-S133 and synaptic associated proteins (PSD95, Syp and Syt-1), and for immunofluorescence staining to detect neuronal activation.

### Behavioral tests

All behavioral studies were performed by two investigators who were blinded to the treatment groups as described previously [8]. Basal responses of mechanical and thermal stimuli were performed 15–20 min prior to each NTG administration or intracerebroventricular injection. Post-treatment responses of mechanical allodynia and thermal hyperalgesia, balance beam walk and negative geotaxis were performed at 2 h after each NTG administration. Before starting to record the data of balance beam walk and negative geotaxis tests, the rats received three days of adaptive training, and the data of basal vestibular function test was recorded on the day before NTG administration.

### Assessment of mechanical allodynia

Tactile sensitivity was measured using the electronic von Frey instrument (Electrovonfrey, 2391, IITC Inc., Woodland Hills, CA, USA) as previous described [21]. Briefly, the pressure probe tip was applied perpendicularly to periorbital region that was over the rostral portion of the eyes, as well as the central area of hind paws surface from a small force value and gradually increasing. The thresholds were automatically recorded when the rat’s head or paw withdrawal was observed. After the initial positive response, repeated stimulations were performed as the same pattern. There was an interval of 5 min between applications. For each rat, three positive responses were obtained to calculate the average threshold.

### Assessment of thermal hyperalgesia

Thermal sensitivity was assessed using Hargreaves radiant heat apparatus (model PL-200, IITC, Taimeng, Chengdu, China) as described previously [8]. Briefly, the rats were placed in a transparent cage to acclimate for 30 min. Then, infrared radiation (intensity: 20%), which caused an abrupt withdraw, was applied to the central part of hind paws. Withdrawal latency was automatically recorded by the device. The average of three records with an interval of 5 min was used to determine the withdrawal latency to the heat.

### Balance beam walk

To evaluate the motor coordination and balance, balance beam walk was performed as previous described [26]. A balance beam of 190 cm in length and 2.5 cm in diameter was placed horizontally at 40 cm above the table. A cushion was placed below to protect the fallen animals. The duration that rats used to pass through the beam was recorded, and the maximum recording time was 15 s (three trials per rat with the interval of 90 s between each trial).

### Negative geotaxis

Negative geotaxis, an automatic unlearned response and directional movement against gravitational cues, is stimulated by the abnormal position of the head and body, initiated by vestibular and postural systems [27, 28]. This test is considered as a diagnostic of vestibular function, reflex development, and motor skills [26]. During this experiment, we used a sponge pad with inclined surface and covered it with a rough towel. As described previously, animals were placed on a 40° inclined surface (ranging from 15° to 70° in most tests) with their head downwards [8]. And the hind paws of rats were about 15 cm away from the top of the slope. The duration for a turn of 180° upward was recorded. The maximum recording time was 15 s (3 trials per rat). And the average of three records with an interval of 1 min was used.

### Quantitative real-time polymerase chain reaction (qPCR)

According to previous studies [8, 26], the VN tissues (localized between − 10 and − 12 mm from bregma) were quickly extracted based on the rat brain atlas of Paxinos and Waston (6th edition) and stored in liquid nitrogen immediately for qPCR. In brief, total RNA from VN tissues was extracted via RNAiso Plus reagent (TaKaRa, Dalian, China) and quantified by the NanoDrop kit (Thermo, USA). Then, the cDNA synthesis was conducted using the PrimeScript™ RT Kit (TaKaRa, Dalian, China). Finally, the mRNA expression of CLR and RAMP1 were detected on a CFX96 Touch thermocycler (Bio-Rad, CA, USA) using SYBR Premix Ex Taq TM II (TaKaRa, Dalian, China). Relative mRNA levels were analyzed using the ΔΔCq method with GAPDH as an internal control. The primer sequences for CLR, RAMP1 and GAPDH (Sangon Biotech, Shanghai, China) were as follows: CLR: F: 5′-CTCATTGTGGTGGCTGTGTTTGC-3′, R: 5′-GCAGGCAGGAAGCAGAGGAAAC-3′; RAMP1: F: 5′-AGGGAAGACTCTGTGGTGTGACTG-3′, R: 5′-GTAGCGGTGGTGGACAGCAATG-3′; GAPDH: F: 5′-ATGACTCTACCCACGGCAAGCT-3′, R: 5′-GGATGCAGGGATGATGTTCT-3′.

### Western blot analysis

As described previously [21], the VN tissues were homogenized in radioimmunoprecipitation assay (RIPA) lysis buffer (Beyotime, Shanghai, China) mixed with protease inhibitor phenylmethylsulfonyl fluoride (PMSF, Beyotime, Shanghai, China) for 1 h at 4 ℃, following centrifugation at 12,000×g in 4 ℃ for 15 min. The bicinchoninic acid (BCA) protein assay kit (Beyotime, Shanghai, China) was used to detect the protein concentration of supernatant. Equal amount of protein samples (30 µg per lane) was loaded on the SDS-PAGE gels (Beyotime, Shanghai, China) for electrophoresis about 2 h, subsequent transferred to the PVDF membranes (Millipore, USA). These membranes were blocked for 2 h in Tris-buffered saline containing Tween 20 (TBST) containing 5% non-fat milk at room temperature and then incubated with primary antibodies overnight at 4 °C. The next day, these membranes were incubated with horseradish peroxidase-conjugated secondary antibodies (goat-anti-rabbit, goat-anti-mouse) diluted in TBST for 1 h at room temperature. The immunoreactive bands were visualized by BeyoECL Plus kit (Beyotime, Shanghai, China), and analyzed by an imaging system (Fusion, Germany). GAPDH and β-actin were used as the internal reference to normalize the relative expression levels of target proteins. The details for each antibody used for western blot are shown in Table 1.

**Table 1:** Antibodies used in western blot and immunofluorescence staining analysis

Antibody	Manufacturer	Host	Dilution
**For Wertern blot analysis**			
CGRP	Abcam, UK	Rabbit	1:3000
PKC	Bioss, China	Rabbit	1:1000
PSD95	Cell signaling technology, USA	Mouse	1:1000
Synaptophysin	Abcam, UK	Rabbit	1:20000
Synaptotagmin-1	Bioss, China	Rabbit	1:1000
p-ERK	Cell signaling technology, USA	Rabbit	1:2000
ERK	Cell signaling technology, USA	Rabbit	1:1000
p-CREB-133	Abcam, UK	Rabbit	1:5000
CREB	Wanleibio, China	Rabbit	1:1000
GAPDH	ZEN BIO, China	Mouse	1:8000
β-actin	ZSGB BIO, China	Mouse	1:1500
Anti-rabbit IgG (HRP)	ZEN BIO, China	Goat	1:5000
Anti-mouse IgG (HRP)	ZEN BIO, China	Goat	1:5000
**For Immunofluorescent staining**			
CGRP	Santa Cruz, USA	Mouse	1:100
CLR	Bioss, China	Rabbit	1:1000
RAMP1	Proteintech, China	Rabbit	1:200
c-Fos	Novus Biologicals, USA	Rabbit	1:5000
NeuN	Abcam, UK	Mouse	1:500
GFAP	Santa Cruz, USA	Mouse	1:200
Iba-1	ThermoFisher, USA	Mouse	1:500
Cy3-conjugatedgoat anti-rabbit IgG	Beyotime, China	Goat	1:500
Alexa Fluor 488 goat anti-mouse IgG	Beyotime, China	Goat	1:500

### Immunofluorescence staining

The tissues were collected 2 h after the final NTG injection to detect the expression of c-Fos, while for other targets, tissues were collected within 12 h. After general anesthesia, the rats were perfused intracardially with 0.1 M PBS 250 mL, followed by 250 mL of 4% paraformaldehyde (PFA). According to previous study [8], the TNC (localized between − 14 and − 16 mm from bregma according to the rat brain atlas of Paxinos and Waston (6th edition)) and VN regions were immediately harvested. The tissues were postfixed in 4% PFA at 4 °C for 24 h, and transferred to dehydration in 20% and then 30% sucrose until tissues sank. The transverse sections of TNC and VN tissues were sectioned at 15 μm thickness on cryostat (Leica, Japan). The slices were permeabilized with 0.3% Triton X-100 for 10 min at 37 °C and blocked with 10% of goat serum (Boster, Wuhan, China) at 37 ℃ for 30 min. Then, the slices were incubated with primary antibodies overnight at 4 ℃. After rinsing 3 times for 15 min in PBS, the sections were incubated with the species-specific fluorophore-labeled secondary antibodies for 90 min at 37 ℃. Finally, the 4′, 6-diamidino-2-phenylinodole (DAPI) (Beyotime, Shanghai, China) was employed to counterstain the nuclei. Images were acquired with a fluorescence confocal microscope (LSM800, Zeiss, Germany) equipped with structured illumination (Zen). The details for primary and secondary antibodies used for immunofluorescence staining are shown in Table 1.

Morphological identifications of TNC and VN were determined as previously described [8]. C-Fos+, CLR+, and RAMP1 + cells, as well as CGRP + fibers were quantified on both sides for TNC and VN from selected serial transverse sections that were collected from the rostral to caudal part. To avoid counting the same cell more than once, each section was separated by at least 250 μm [8]. The number of target cells was assessed with x200 magnification through the optical fractionator method, while the fluorescence signal intensity of CGRP + fibers was determined using Image-pro Plus 6.2 software (Bethesda, MD, USA) (*n* = 6 rats per group, 5 images per rat). 

### Transmission electron microscopy

After general anesthesia, rats were intracardially perfused with 2.5% glutaraldehyde as previous described [21]. The VN tissues were collected and fixed by 4% glutaraldehyde at 4℃ overnight. Next, the VN tissues were cut into 1mm^3^ pieces and rinsed with PBS for 30 min. The samples were post-fixed with 1% osmium tetroxide for 2 h, followed by dehydrating in a graded series of ethanol (50%, 70%, 90%, 100% and 100%, each concentration for 10 min) and transferring to 100% propylene oxide for 15 min, and then embedded in Epon-Araldite resin (Canemco & Marivac, Lakefield, Quebec, Canada). Ultrathin Sects. (80–90 nm) were prepared with an ultramicrotome (Reichert-Jung, Inc., Cambridge, UK). Then sections were washed twice with distilled water, and counterstained with 2% uranyl acetate containing with lead citrate for 45 min. Sections were visualized on a transmission electron microscope (JEM-1400 PLUS, JEOL Ltd., Japan).

The thickness of the postsynaptic density (PSD) and length of synaptic activity zone, as well as the curvature of synaptic interface were measured in accordance with previous protocol [29]. The observer, who was blinded to the experiment groups, analyzed images using Image-pro Plus 6.2 software (Bethesda, MD, USA). Each group contained 3 rats, and 3 ultrathin sections were obtained from each rat. Five images per section were obtained to estimate the average values.

### Golgi-cox staining

The morphological changes of neuronal dendritic spines were determined through Golgi-cox staining. An FD Rapid Golgi Stain Kit™ (FD Neuro Technologies Columbia, MD, USA) was used according to the manufacturer’s instructions [29]. After collecting VN tissues, samples were immediately immersed in the Solution A/B for 14 d and then transferred to the Solution C for 3 d at room temperature protected from light. Samples were cut as a series of 150 μm coronal sections via a vibratome (Leica VT 1200 S, Japan). The staining process was performed according to the manufacturer’s instructions. In brief, sections were stained with the mixed working solution (Solution D: Solution E: double-distilled water = 1:1:2) for 10 min. After rinsing in double-distilled water for 2 times, sections were then dehydrated in a graded series of ethanol (50%, 75%, 95% and 100%, 4 min each concentration) and xylene (3 times, 4 min each time) before a coverslip with Permount™ Mounting Medium (Fisher Scientific Co., MA USA) was placed.

Glutamatergic and GABAergic neurons are two primary neural components of VN, and glutamatergic neurons were recently considered to act as the fundamental role in controlling posturo-locomotor behaviors [30]. Thus, the excitatory pyramidal neurons in the VN region obtained under a microscope (Axio Imager A2, Zeiss, German) were selected for dendritic spines analysis according to previous protocol [31]. The blinded investigator analyzed images using Image-pro Plus 6.2 software (Bethesda, MD, USA) (*n* = 3 rats/group, 5 images for each rat).

### Statistical analysis

All the data were presented as the mean ± SD. SPSS 20.0 software (SPSS Inc., IBM, USA) and GraphPad Prism version 8.0 (GraphPad Software Inc, CA, USA) was used for statistical analysis and graph generation. Two-tailed Student’s *t*-test was performed for statistical comparisons between two groups. One-way ANOVA followed by post hoc analysis with the Dunnett’s test was used for statistical comparisons among groups. Two-way ANOVA with the Bonferroni post hoc test was used for behavioral data analysis. Mann-Whitney *U* test was performed for the nonparametric analysis. The value of *p* < 0.05 was considered statistically significant.

## Results

### Repeated NTG administration induced mechanical allodynia, thermal hyperalgesia and vestibular dysfunction

**Fig. 1:**
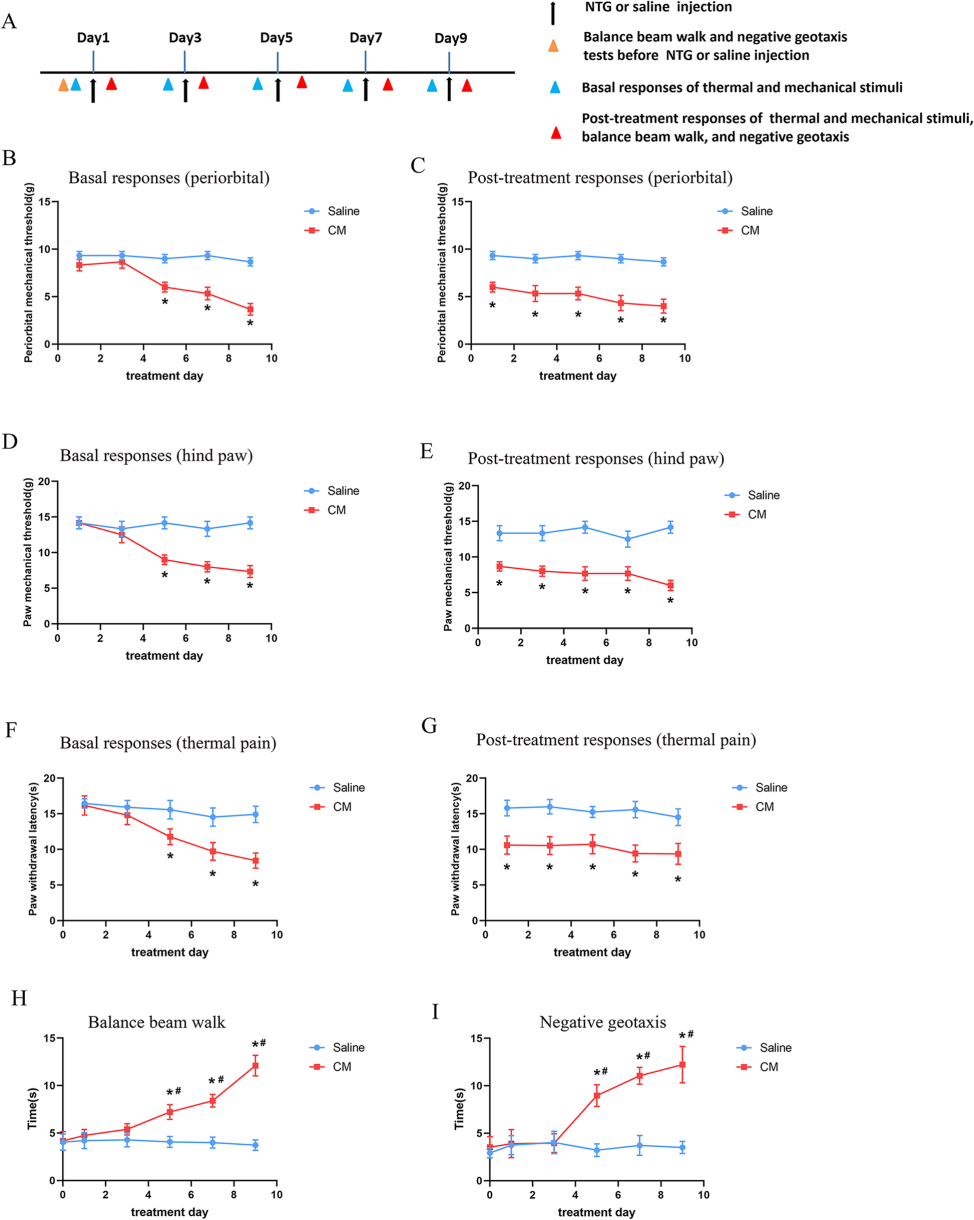
Recurrent NTG administration induced mechanical allodynia, thermal hyperalgesia, and vestibular dysfunction. **A.** Timeline of behavior studies’ protocol. **B-G**. Basal and post-treatment responses of periorbital (**B, C**) and hind paw (**D, E**) mechanical and thermal thresholds (**F, G**) after NTG injection. Balance beam walk (**H**) and geotaxis reflex test (**I**) in each group. n = 6/group. Results are mean ± SD. Two-way analysis of variance (ANOVA), Bonferroni; *p < 0.05 compared with saline group, #*p* < 0.05 compared with before NTG injection

**Fig. 2:**
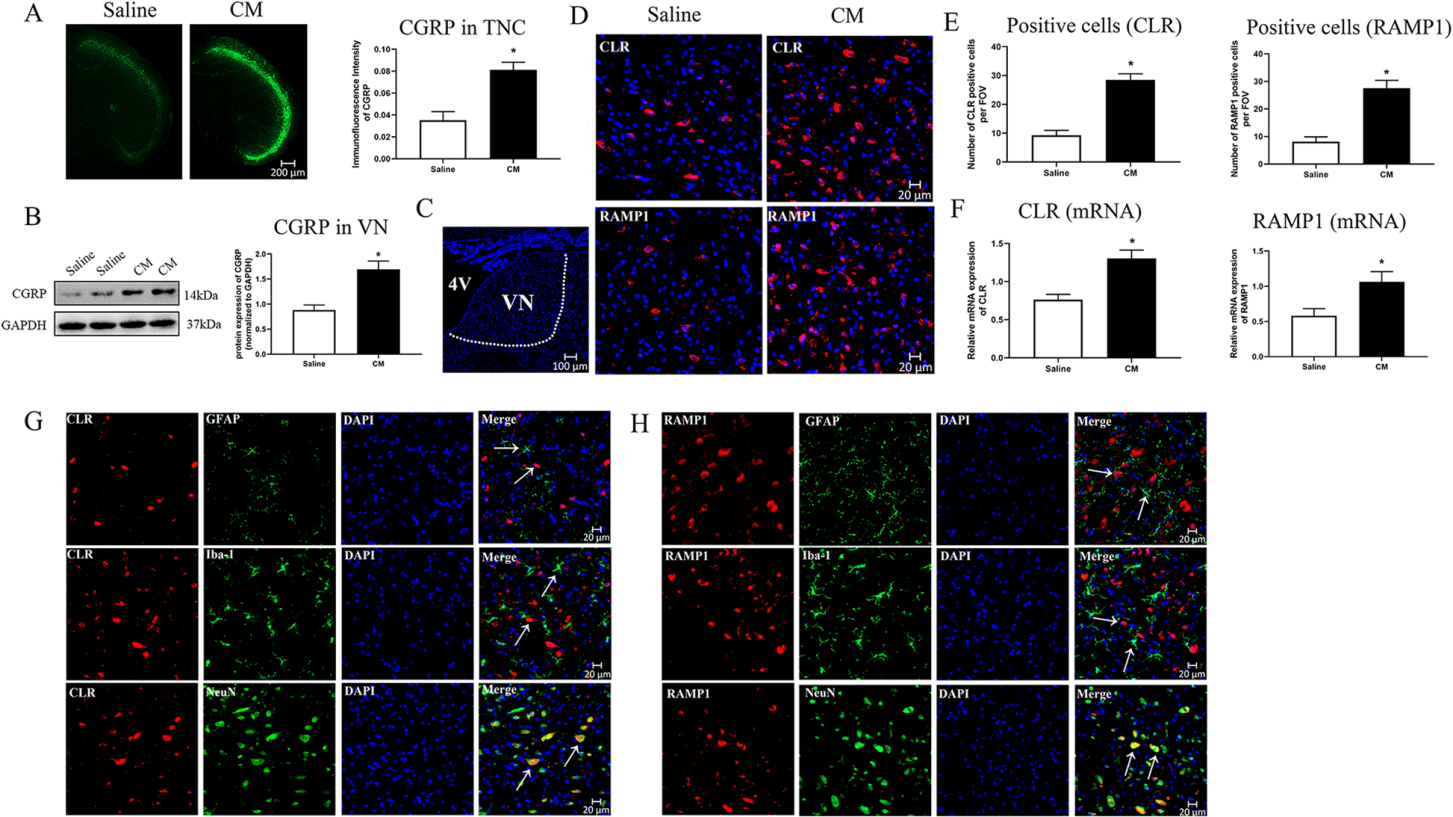
Increased expression levels of calcitonin gene-related peptide (CGRP) and its receptor components in the vestibular nucleus (VN) after CM. **A** Representative micrographs and quantitative analysis of CGRP positive (green) fibers in the trigeminal nucleus caudalis (TNC). *n* = 6/group. **B** Representative western blots bands and densitometric quantification of CGRP in VN. *n* = 6/group. **C** Schematic illustration of brain tissue shows the VN region depicted with a white dotted line. Representative micrographs (**D**) and quantitative analysis (**E**) of calcitonin receptor-like receptor (CLR) and receptor activity modifying protein 1 (RAMP1) in VN. *n* = 6/group. **F** The mRNA levels of CLR and RAMP1 in VN. *n* = 6/group. **G H** Representative microphotographs of co-immunofluorescence staining of CLR (red) and RAMP1 (red) with astrocytes (GFAP, green), microglia (Iba-1, green), and neurons (NeuN, green) in VN after CM. The nuclei were stained with DAPI (blue). *n* = 3/group. Results are mean ± SD. FOV = 1.02 × 10^6^ µm^3^. One-way ANOVA, Dunnett; *p < 0.05 compared with saline group. 4 V: the fourth ventricle

We used intermittent NTG administration for 9 days as a model of CM. The model produces mechanical allodynia and thermal hyperalgesia that lasts for 7 days after final treatment [32]. The thresholds for mechanical stimulation, as well as the latencies to noxious heat were significantly reduced in a time dependent manner on day 5, 7 and 9 in NTG-treated animals when compared with saline -treated animals (*p* < 0.05; Fig. 1B and D F). Acute hypersensitivity was evident by sustained reduced thresholds for withdrawal from mechanical (*p* < 0.05; Fig. 1 C and 1E) and thermal stimulation (Fig. 1G). Consistent with our previous study, chronic injection of NTG produced marked vestibular dysfunction [8]. In line with the development of tactile and thermal hypersensitivity, NTG administration significantly extended the time that rats spent traversing the balance beam (*p* < 0.05; Fig. 1 H) and that turn to 180° upward in negative geotaxis test (*p* < 0.05; Fig. 1I) on day 5, 7 and 9 when compared with the saline group and before injection data. Furthermore, the elevated expression of CGRP in the superficial layers of TNC has been considered as a key index to the development of central sensitization after CM [10, 33]. Immunofluorescence staining showed that the density of CGRP-immunoreactive fibers in TNC was significantly increased in CM group compared to saline group (*p* < 0.05; Fig. 2 A). The above data all indicated that we established a reliable rat model of CM that had vestibular dysfunction, which could be used in the following experiments.

### Endogenous CGRP, CLR and RAMP1 were upregulated in VN after CM

First, we examined whether endogenous CGRP expression in VN would change in response to repeated NTG administration. We evaluated expression of CGRP by western blot analysis in CM and saline group. The protein level of endogenous CGRP was significantly increased after CM compared to saline group (*p* < 0.05; Fig. 2B). Given that CGRP1 receptor complex has two functional components, we then evaluated expression levels of CLR and RAMP1 by immunofluorescence staining and qPCR in CM rats. Compared to the saline group, the number of CLR+ (*p* < 0.05; Fig. 2D upper panel and 2E left) and RAMP1+ (*p* < 0.05; Fig. 2D lower panel and 2E right) cells increased significantly in CM rats. Similar changes have been observed in the mRNA levels of CLR and RAMP1 (Fig. 2 F), indicating that these two receptor components were activated in VN after CM. Therefore, CGRP1 receptor specific antagonist, BIBN4096BS, was selected as the main treatment after CM.

Furthermore, double immunofluorescence staining showed that CLR (Fig. 2G lower panel) and RAMP1 (Fig. 2 H lower panel) were abundantly expressed in neurons located VN areas (Fig. 2 C) of CM rats. By contrast, CLR and RAMP1 seemed to have minor expression level in astrocytes (Fig. 2G H upper panel) and microglia (Fig. 2G H middle panel) in VN of CM rats (GFAP for astrocytes, Iba1 for microglia, and NeuN for neurons).

### Blockage of CGRP1 receptor with BIBN4096BS attenuated NTG-induced mechanical allodynia, thermal hyperalgesia and vestibular dysfunction

**Fig. 3:**
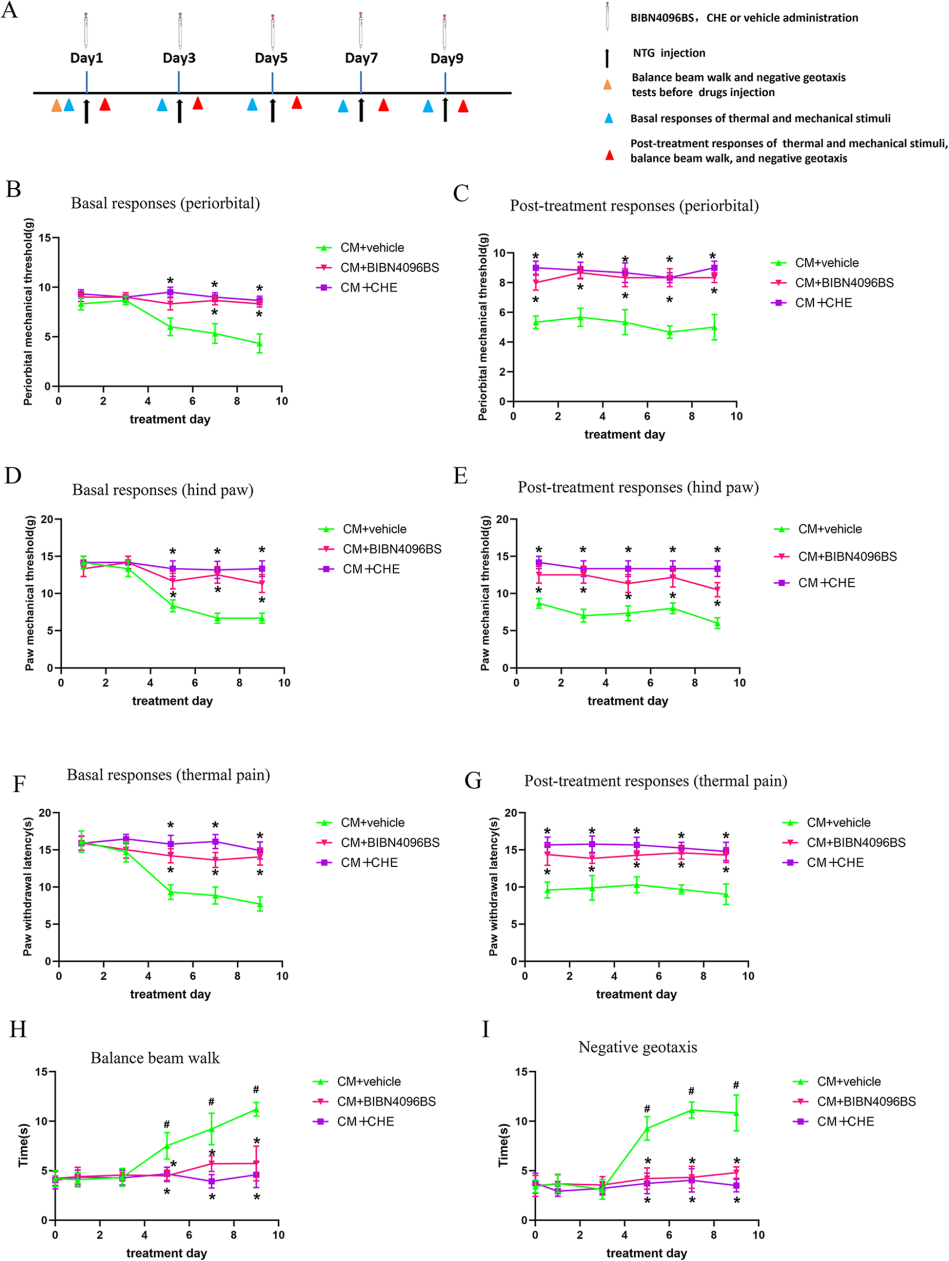
BIBN4096BS and chelerythrine chloride (CHE) treatment ameliorated mechanical allodynia, thermal hyperalgesia and vestibular dysfunction after CM. **A** Timeline for the treatment study. BIBN4096BS and CHE (PKC inhibitor) treatment ameliorated the decreased mechanical (**B-E**) and thermal (**F-G**) thresholds after CM. BIBN4096BS and CHE treatment alleviated vestibular dysfunction (**H-I**) after CM. *n* = 6/group. Results are mean ± SD. Two-way ANOVA, Bonferroni; *p < 0.05 compared with CM + vehicle group, #*p* < 0.05 compared with before NTG injection

To further investigate the role of CGRP1 receptor in the development of mechanical allodynia, thermal hyperalgesia and vestibular dysfunction after CM, we administered CGRP1 receptor antagonist BIBN4096BS (0.1 µg/day), a noncompetitive antagonist, to the lateral ventricle prior to each NTG injection. Single treatment with BIBN4096BS markedly attenuated acute mechanical allodynia and thermal hyperalgesia compared to CM + vehicle group (*p* < 0.05; Fig. 3 C, E, and G). Multiple treatments with BIBN4096BS significantly increased basal mechanical and thermal pain thresholds when compared with CM + vehicle group (*p* < 0.05; Fig. 3B, D, and F). BIBN4096BS treatment significantly reversed NTG-induced vestibular dysfunction, as evidenced by less time in traversing the balance beam and negative geotaxis test compared to CM + vehicle group (*p* < 0.05; Fig. 3 H and I).

### Inhibiting CGRP1 receptor with BIBN4096BS ameliorated overexpression of synaptic associated proteins

**Fig. 4:**
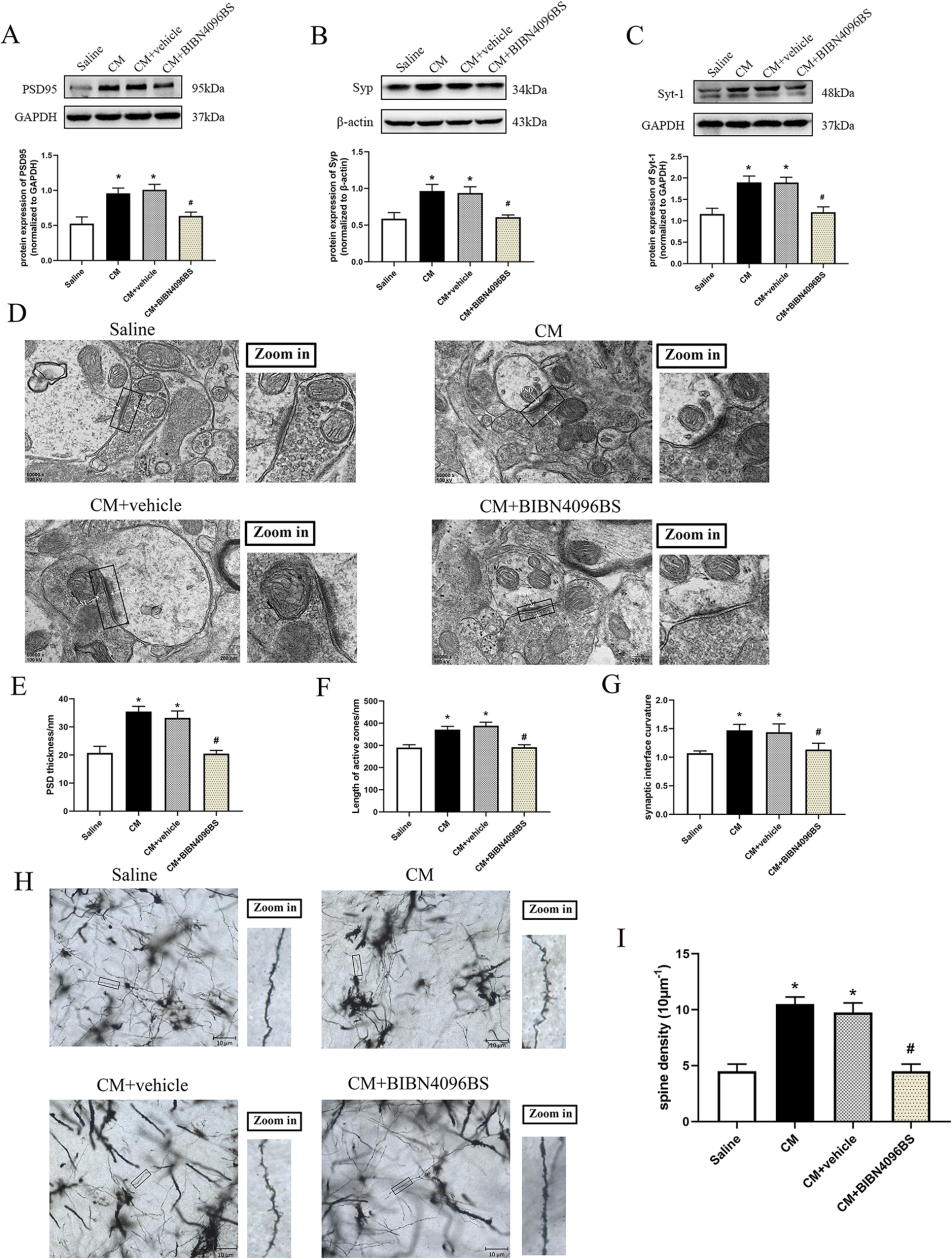
BIBN4096BS treatment reduced overexpression of synaptic associated proteins and restored the aberrant synaptic ultrastructure in VN after CM. Representative western blot bands and densitometric quantification of PSD-95 (**A**), Syp (**B**) and Syt-1 (**C**) after CM. *n* = 6/group. **D** Representative images of the synaptic ultrastructure in VN after CM. Quantitative analysis of PSD thickness (**E**), length of synaptic activity zone (**F**), and curvature of synaptic interface (**G**) after CM. *n* = 3/group. Representative images (**H**) and quantitative analysis (**I**) of the density of dendritic spines in VN after CM. *n* = 3/group. Results are mean ± SD. One-way ANOVA, Dunnett; *p < 0.05 compared with saline group, #*p* < 0.05 compared with CM + vehicle group

In order to explore the involvement of enhanced synaptic transmission in VN after CM, and the regulating role of BIBN4096BS on synaptic transmission efficiency in CM, we first analyzed several key synaptic associated proteins (post-synaptic density protein 95: PSD95; synaptophysin: Syp; synaptotagmin-1: Syt-1) in VN by western blot analysis. PSD95 and Syp are representative makers of postsynaptic and presynaptic respectively. And Syt-1 is an important Ca^2+^-sensor that strengthens synaptic connections [31]. While the expressions of PSD-95, Syp and Syt-1 were significantly increased in the CM compared to saline group (*p* < 0.05; Fig. 4 A-C). Inhibiting CGRP1 receptor with BIBN4096BS markedly decreased the expression levels of PSD-95, Syp and Syt-1 compared to the CM + vehicle group (*p* < 0.05; Fig. 4 A-C).

### Inhibiting CGRP1 receptor with BIBN4096BS restored the aberrant synaptic ultrastructure and the density of neuronal dendritic spines

The structural alterations of synapses are critical to synaptic transmission and synaptic plasticity [34]. Thus, the thickness of PSD, length of synaptic activity zone, and curvature of synaptic interface were selected as morphological indicators of synapses in this study [21]. When compared with saline group, CM significantly increased the PSD thickness (*p* < 0.05; Fig. 4D and E), length of synaptic activity zone (*p* < 0.05; Fig. 4D and F), and synaptic interface curvature (*p* < 0.05; Fig. 4D and G). After multiple BIBN4096BS treatments, these morphological alternations were significantly alleviated, and the morphological characteristics of synapses were comparable as those in saline group (Fig. 4D-G).

Furthermore, as described in previous study [31], we detected the alternation of dendritic spines in VN, which is considered as the major postsynaptic sites for excitatory input [35]. Golgi-cox staining showed more abundant dendritic spines in CM and CM + vehicle group compared to saline group (*p* < 0.05; Fig. 4 H and I). Multiple BIBN4096BS treatments significantly decreased the density of dendritic spines (*p* < 0.05; Fig. 4 H and I). These results suggested an enhanced synaptic transmission in VN after CM, while BIBN4096BS treatment could significantly abolish this enhancement.

### Inhibiting CGRP1 receptor with BIBN4096BS regulated the PKC/ERK/CREB signal and attenuated neuronal activation in VN after CM

**Fig. 5:**
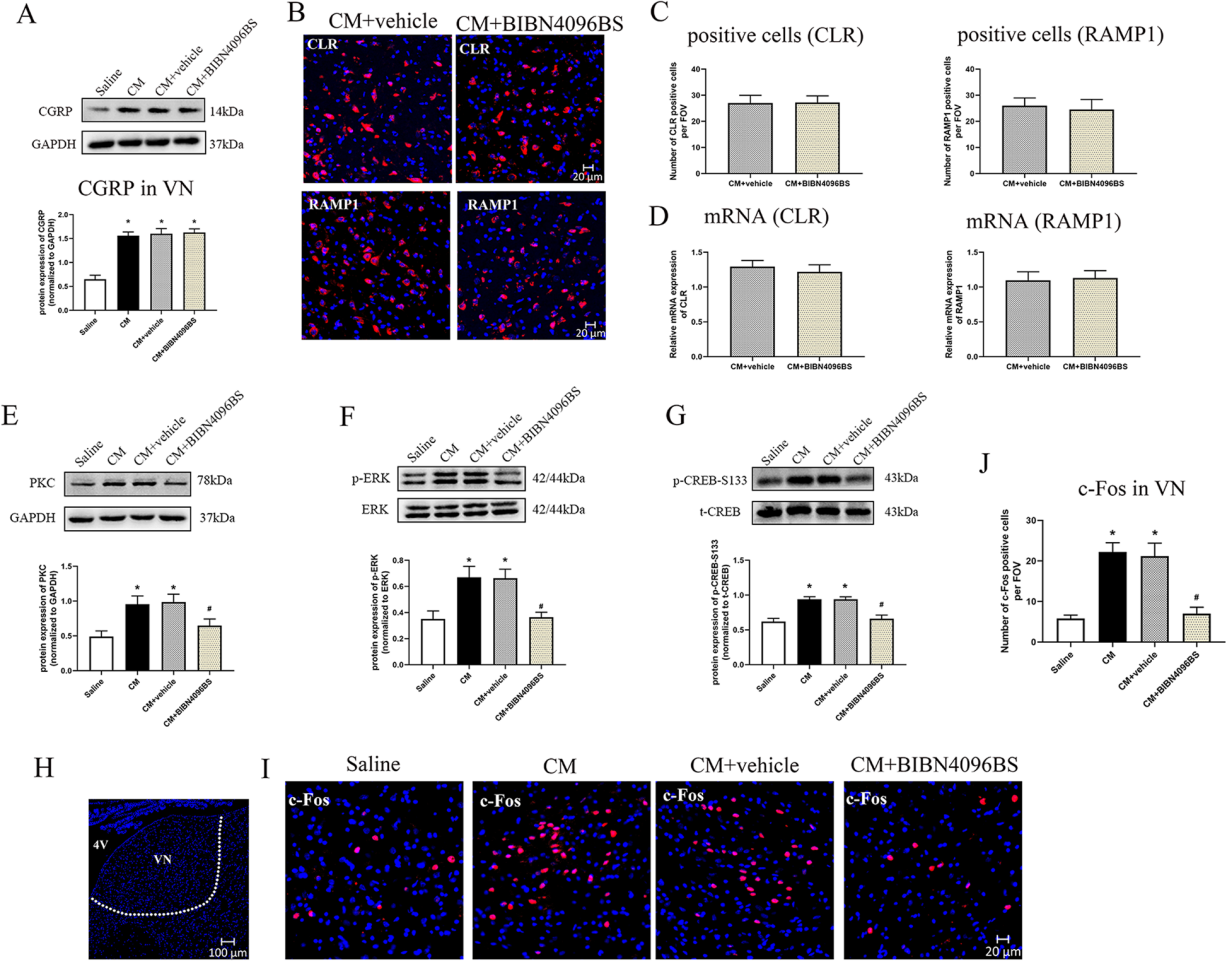
BIBN4096BS treatment regulated the PKC/ERK/CREB signal and attenuated neuronal activation in VN after CM. **A** Representative western blot bands and densitometric quantification of CGRP in VN. *n* = 6/group. Representative micrographs (**B**) and quantitative analysis (**C**) of CLR and RAMP1 positive staining cells in VN. *n* = 6/group. **D** The mRNA levels of CLR and RAMP1 in VN. *n* = 6/group. Representative western blot bands and densitometric quantification of PKC (**E**), p-ERK (**F**) and p-CREB-S133 (**G**) in VN after CM. *n* = 6/group. The white dotted line frame indicated the VN area used for analysis (**H**). Representative micrographs (**I**) and quantitative analysis (**J**) of c-Fos staining in VN. The nuclei were stained with DAPI (blue). *n* = 6/group. Results are mean ± SD. FOV = 1.02 × 10^6^ µm^3^. One-way ANOVA, Dunnett; *p < 0.05 compared with saline group, #*p* < 0.05 compared with CM + vehicle group. 4 V: the fourth ventricle

In vivo and in vitro studies show that the PKC/ERK/CREB pathway plays an important role in CGRP related pain transmission and central sensitization [19, 20]. However, whether the PKC/ERK/CREB signaling was a potential pathway involved in CGRP1 receptor-mediated regulation of synaptic transmission in VN after CM remains unclear. The endogenous expressions of PKC, phosphorylated ERK and CREB-S133 were significantly elevated in CM group compared to saline group (*p* < 0.05; Fig. 5E-G). The addition of BIBN4096BS did not alter CGRP, CLR and RAMP1 expressions in VN after CM (Fig. 5 A-D). The expression of PKC, phosphorylation of ERK and CREB-S133 were significantly reduced in BIBN4096BS treated CM rats compared to vehicle treated CM rats (*p* < 0.05; Fig. 5E-G).

**Fig. 6:**
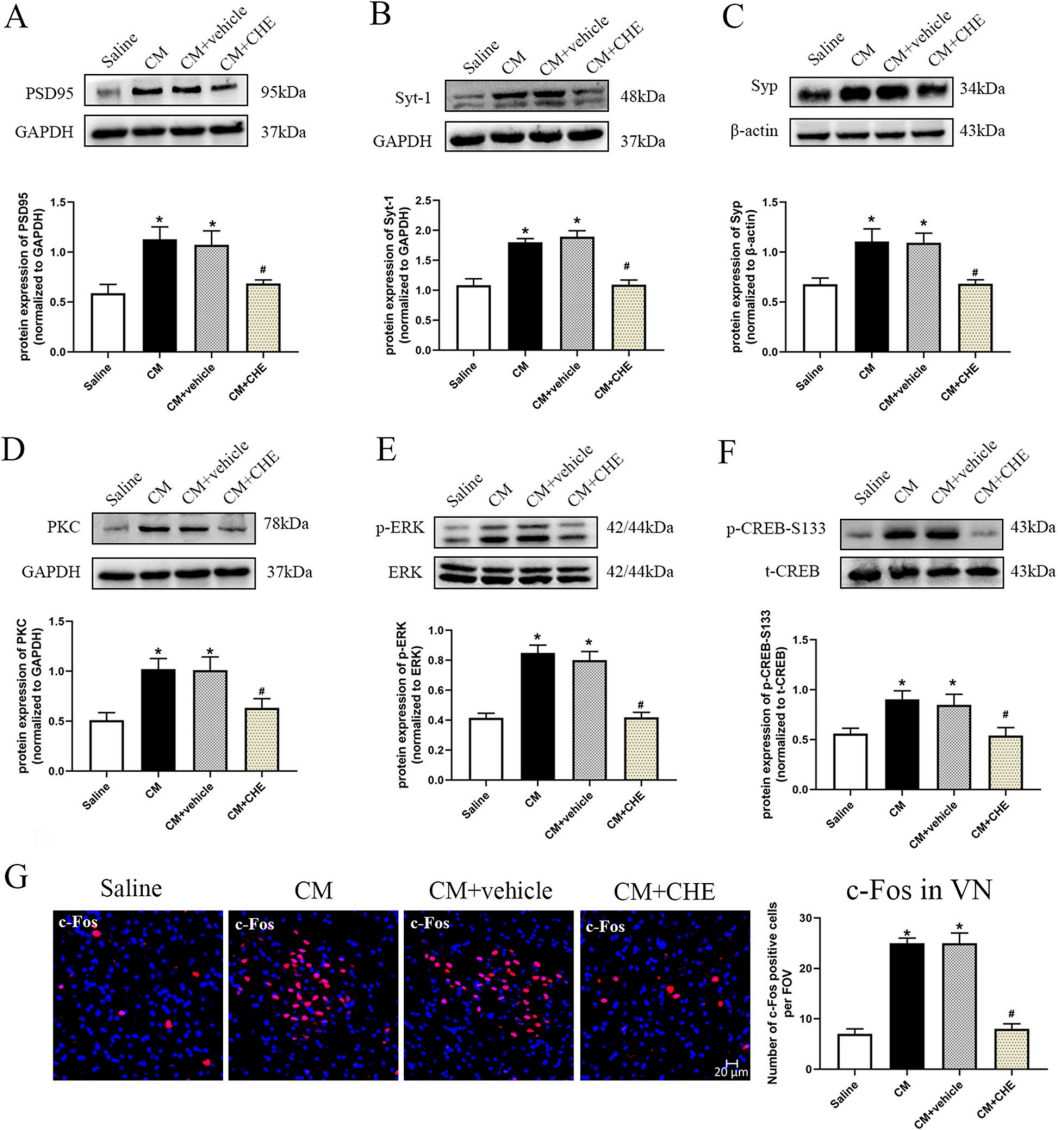
Inhibition of PKC activity suppressed NTG-induced overexpression of synaptic associated proteins, reduced downstream phosphorylated ERK and CREB-S133 levels and inhibited neuronal activation after CM. Representative western blot bands and densitometric quantification of PSD-95 (**A**), Syt-1 (**B**), Syp (**C**), PKC (**D**), p-ERK (**E**) and p-CREB-133 (**F**) after CM. *n* = 6/group. **G**. Representative micrographs and quantitative analysis of c-Fos staining in VN. The nuclei were stained with DAPI (blue). *n* = 6/group. Results are mean ± SD. FOV = 1.02 × 10^6^ µm^3^. One-way ANOVA, Dunnett; *p < 0.05 compared with saline group, #*p* < 0.05 compared with CM + vehicle group

Administration of CHE, the specific PKC inhibitor, to CM rats showed similar behavior improvements as CM + BIBN4096BS group when compared with CM + vehicle group (Fig. 3B-I). Treatment with CHE markedly decreased the expression levels of PSD-95, Syt-1 and Syp compared to the CM + vehicle group (p < 0.05; Fig. 6 A-C). The expression of PKC, phosphorylation of ERK and CREB-S133 were significantly reduced in CHE treated CM rats compared to CM and CM + vehicle (*p* < 0.05; Fig. 6D-F). These results support the notion that CGRP may regulate synaptic transmission through CGRP1 receptor/PKC/ERK/CREB signal.

C-Fos has been demonstrated as a classic marker for detecting neuronal activation after noxious stimulation [36]. Quantitative analysis of immunofluorescence staining found a greater density of c-Fos positive staining neurons in VN after CM, and this effect was abolished by BIBN4096BS (*p* < 0.05; Fig. 5I-J). PKC inhibition showed similar tendency as BIBN4096BS administration (Fig. 6G).

## Discussion

Multiple lines of evidence show that migraine and vestibular symptoms are frequently encountered clinically [2, 4, 37]. The rate of disability is high, and there is currently a lack of effective prophylactic treatment [38]. The exact pathophysiology of vestibular symptoms in migraine is incompletely understood but could include anatomical pathways and neurochemical modulation between TNC and VN [8, 39]. CGRP or CGRP1 receptor antibodies and antagonists have proved to appreciably alleviate headache severity and frequency in both of episodic and chronic migraine patients [9]. Our previous study found that increased expression of CGRP was positively associated with neuronal activation in VN, as well as vestibular dysfunction after CM [8]. To date, whether CGRP1 receptor involves in the development of vestibular sensitization, and how CGRP regulates neuronal activation in VN after CM remains largely unknown. Thus, the present study was to investigate whether CGRP1 receptor contributes to vestibular dysfunction after CM by regulating synaptic transmission in VN.

Our results showed that CGRP and two major components of its receptor, CLR and RAMP1, were significantly elevated in VN after CM. CLR and RAMP1 were primarily co-expressed with neurons in VN. Multiple treatments with CGRP1 receptor selective antagonist, BIBN4096BS, alleviated mechanical allodynia, thermal hyperalgesia and vestibular dysfunction induced by intermittent administration of NTG, and this process was associated with the downregulation of synaptic associated proteins, restoration of aberrant morphological characteristics of synapses, and suppression of neuronal activation in VN after CM. Inhibiting CGRP1 receptor with BIBN4096BS decreased the overexpression of PKC, and phosphorylation of ERK and CREB-S133 in VN after CM. Taken together, our data suggests the therapeutic potentials of BIBN4096BS to restore vestibular dysfunction in patients with CM.

Consistent with our previous study, we established a reliable CM model that had vestibular dysfunction via systematic NTG administration [8]. Each NTG administration caused several behavior signs of allodynia and hyperalgesia, mimicking the acute attack of migraine. While repeated NTG administration induced the pronounced and sustained allodynia and hyperalgesia as found in most of CM patients [10, 32]. Unlike the lowered pain thresholds, significant vestibular dysfunction was not observed after the initial NTG injection, and vestibular function was exaggerated after the third NTG injection. These phenomena were consistent with those reported in clinical-based studies: the development of vestibular symptoms, including motion intolerance, often lagged several years behind headache in migraine patients; patients with chronic migraine experienced more frequent vestibular symptoms than patients with episodic migraine [2, 40, 41]. Additionally, migraine is more prevalent in women than in men, which are mainly due to the role of estrogen [42, 43]. In fact, the regulation of pain by estrogen is very complicated. The level and stability of estrogen can affect its modulation of pain, including aggravating or relieving [44]. The main trigger factors of menstrual-related migraine seem to be the withdrawal of estrogen, and reducing the magnitude of decline in estrogen concentration can prevent menstrual-related migraine [43, 45]. Studies have found that endogenous changes in the concentration of estrogen and progesterone in premenstrual syndrome or the use of exogenous hormones (such as oral contraceptives) may cause vertigo [46]. In order to avoid the influence of estrogen, we chose male mice to establish the model. Given the significant sex differences in the prevalence of migraine, the sex dimorphism of CGRP1 receptor involvement in pathogenesis of migraine-related dizziness requires further exploration.

A plethora of studies have demonstrated the critical role of CGRP in the trigeminovascular system that are involved in migraine pathology [47]. Abundant expression of CGRP in laminae I and II of the TNC has been reported as an important indicator for central sensitization of CM, which is responsible for the development of spontaneous cephalic cutaneous and extracephalic allodynia [5, 21]. Consistently, this study also observed the elevated expression of CGRP in TNC and VN after CM. In addition, reducing CGRP expression in VN attenuated excitability of neurons and reversed the vestibular dysfunction induced by NTG administration [8]. These data indicated that elevated CGRP level in VN might be an index to vestibular sensitization in CM rats. The various effects of CGRP act primarily through CGRP1 receptor, which comprising CLR and RAMP1 [47, 48]. In preclinical animal models and clinical trials, “gepants” are proved to have high affinity for CGRP1 receptor [48]. To make it easier to translate our findings for clinical application, the present study applied BIBN4096BS (namely: olcegepant) as the treatment regimen [48, 49]. However, another GPCR, the calcitonin receptor (CTR), which is closely-related to CLR, together with RAMP1 (namely: AMY_1_ receptor) recently has been reported to have high affinity for both of CGRP and amylin [48]. Additionally, adrenomedullin, an amylin analogue, is demonstrated to effectively provoke migraine-like headache in migraine patients [50]. Less is known about the nociceptive role of AMY_1_ receptor in migraine pathology and the clinical significance of the interaction between CGRP and AMY_1_ receptor. Thus, we selected CGRP1 receptor as the primary receptor for the following studies.

CGRP1 receptor are broadly expressed in the mammalian CNS [5, 51]. Considering the limited passage of the gepants (2%) across the blood–brain barrier, BIBN4096BS was administrated via intracerebroventricular route to explore the central action of CGRP1 receptor [47]. We found that antagonizing CGRP1 receptor significantly abolished behavioral signs of mechanical allodynia, thermal hyperalgesia and vestibular dysfunction in CM rats, and these were closely related with the neuronal activation in VN. But how CGRP modulates the neuronal activation in VN after CM is still unclear. According to the immunofluorescence staining, CLR and RAMP1 were both primarily co-expressed with neurons, implying that CGRP might exert its effect mainly through the neuronal CGRP1 receptor. In vivo and in vitro studies demonstrated that exogenous CGRP can enhance the neural synaptic transmission in spinal cord, anterior cingulate cortex and insular cortex, and that could be notably blocked by BIBN4096BS [15, 16, 52, 53]. Therefore, we proposed that CGRP could modulate the synaptic transmission efficiency via CGRP1 receptor in VN after CM. We first examined the expression of several synaptic associated proteins, including PSD95, Syp and Syt-1, in VN after CM. PSD95 is a structural protein located on the postsynaptic membrane, implicated in synaptic maturation, strengthening, and plasticity [54]. Syp, a major integral membrane protein of synaptic vesicles, is necessary for vesicle formation and exocytosis, which can regulate the efficiency of synaptic transmission by influencing the synaptic structure and the release of neurotransmitters [55, 56]. Syt-1, acting as a Ca^2+^ sensor at neuronal synapses, is a marker of synaptic transmission, which synchronizes neurotransmitter release with Ca^2+^ influx during action potential firing and mediates ultrafast exocytosis of synaptic vesicles [57, 58]. Our study found that the expression of synaptic associated proteins was significantly increased after CM, while multiple treatments of BIBN4096BS markedly downregulated their expression. Moreover, we detected the synaptic ultrastructure and the density of neuronal dendritic spines of VN neurons in CM rats, which are closely related to synaptic transmission efficiency, to further confirm that CGRP/CGRP1 receptor involves in the regulation of synaptic transmission in VN after CM.

We further explored downstream orchestrators of CGRP1 receptor inhibition to determine potential mechanisms for neuronal activation. Activation of the glutamate N-methyl-D-aspartate (NMDA) receptors is a hallmark to the development and maintenance of central sensitization in chronic pain, including migraine [10]. Incubation with CGRP or activation of CGRP1 receptor significantly promotes NMDA receptors-mediated excitatory potentials in central amygdala [10, 59]. The downstream activation of PKC induces phosphorylation of NMDA receptors and increases calcium inputs which further enhances the excitability of dorsal horn neuron, thus maintaining a state of central sensitization [10, 19]. Similar to the previous results, we found that the expression levels of PKC, phosphorylated ERK and CREB-S133 were significantly increased in CM rats. The effects of NTG administration on PKC expression and phosphorylation of ERK and CREB-S133 were reversed by BIBN4096BS. Additionally, inhibition of PKC with CHE reduced the overexpression of synaptic associated proteins, p-ERK and p-CREB-S133 caused by CM, and inhibited the activation of neurons in the VN of CM rats. These results suggested that CGRP may improve synaptic transmission and neuron activation of VN through CGRP1 receptor/PKC/ERK/CREB in CM rats.

In neuronal circuits, the vast majority of excitatory synaptic transmission occurs at the postsynaptic dendritic spines [60]. In our studies, we found more abundant dendritic spines in CM group compared to saline group. And multiple BIBN4096BS treatments significantly decreased the density of dendritic spines in VN. Protein kinase C (PKC)-dependent mechanisms can promote synaptic function in the mature brain. Studies found that the activation of protein kinase C (PKC) ε and α has been linked to synaptogenesis. And PKCε/α activator bryostatin can increase dendritic spine density of pyramidal neurons in hippocampal CA1 region of aged rats [61]. In mature mouse hippocampal neuron cultures, PKC activator bryostatin increased the proportion of mushroom dendritic spines in total spine density, enhanced synaptic transmission [62]. As well, atypical protein kinase C (aPKC), a subgroup of PKC, can promote increased dendritic spine density in hippocampal neurons through p190 RhoGAP and RhoA pathway [63, 64]. In our research, we found that inhibiting PKC with chelerythrine chloride (CHE) ameliorated the overexpression of synapse-associated proteins. Therefore, we speculate that the activation of PKC pathway can also cause an increase in the density of neuronal dendritic spines at the VN site. More direct evidence is needed in our future studies.

According to previous research, under physiological conditions, the GABAergic neurons that project to the vestibular nucleus are mainly located in spinal trigeminal nucleus orails (Sp5O) and a small amount of spinal trigeminal nucleus interpolaris (Sp5I), and the glutamatergic neurons that project to VN are mainly located at Sp5O, Sp5I and spinal trigeminal nucleus candalis (Sp5C or TNC). GABA immunoreactive neurons account for about 15% of trigeminal vestibular neurons, and glutamatergic neurons account for about 37% of the trigeminal vestibular neurons [39]. Our previous study found that CM induced neuronal activation in the TNC and VN, and TNC-projecting VN neurons were activated after CM [8]. As well, glutamatergic neurons of VN, the excitatory neurons, were recently considered to act as the fundamental role in controlling posturo-locomotor behaviors [30]. However, we currently have no direct evidence for the type of neurons activated at the VN site in CM rats. We will explore it more deeply in future research.

There are several limitations in this study. Although CGRP1 receptor is mainly expressed in neurons of VN areas, we cannot completely exclude that the neuroprotective effects exerted by CGRP1 receptor inhibition may involve glia in CM rats. Also, we are focusing on PKC/ERK/CREB signaling, but other mechanisms require further investigation. Then, we didn’t perform the basal vestibular function test before each NTG administration. Although we speculate that vestibular dysfunction in migraine patients may be a progressive rather than acute process, the data of the vestibular function test before each NTG administration may help us understand the development of the disease comprehensively. Our research ignored the study of three synaptic associated proteins in the synaptic subcellular fractions, and we will pay attention to this part in the future research. In our study, BIBN4096BS and protein kinase C (PKC) inhibitor chelerythrine chloride (CHE) were administered intracerebroventricularly, rather than local microinjection into the VN. Therefore, we cannot completely rule out effects of these drugs on other brain regions. Last, we only explored the effects of morphological alternations on synaptic transmission. Future studies on electrophysiology are needed to examine the effects on synaptic function.

## Conclusions

In conclusion, our results demonstrated that CGRP/CGRP1 receptor involved in the morphological alternation of synapses in VN and influenced vestibular function in an experimental CM rat model, at least partly, through PKC/ERK/CREB pathway. Most importantly, we also demonstrated that BIBN4096BS, a specific CGRP1 receptor antagonist, restored the synaptic ultrastructure, attenuated enhancement of neuronal activity and alleviated mechanical allodynia, thermal hyperalgesia and vestibular dysfunction in CM rats. Our findings may provide new strategies for the treatment of migraine patients with vestibular symptoms.

## Data Availability

The data used and analyzed in this article are available from on reasonable request.

## Abbreviations

ANOVA: Analysis of variance
ARRIVE: Animal Research Reporting of In Vivo Experiments
CGRP: Calcitonin Gene-related Peptide
CLR: Calcitonin Receptor-like Receptor
CREB: cAMP Response Element-binding Protein
CM: Chronic Migraine
CHE: Chelerythrine Chloride
ERK: Extracellular Signal Regulated Kinase
FOV: Field of View
GPCR: G-protein-coupled Receptor
NMDA: Glutamate N-methyl-D-aspartate
NTG: Nitroglycerin
PKC: Protein Kinase C
PSD95: Post-synaptic Density Protein 95
PSD: Postsynaptic Density
qPCR: Quantitative Real-time Polymerase Chain Reaction
RAMP1: Receptor Activity Modifying Protein 1
Syp: Synaptophysin
Syt-1: Synaptotagmin-1
Sp5O: trigeminal nucleus orails
Sp5I: trigeminal nucleus interpolaris
Sp5C: trigeminal nucleus candalis
TNC: Trigeminal Nucleus Caudalis
VN: Vestibular Nucleus
